# Evaluation and comparison of errors on nursing notes created by online and offline speech recognition technology and handwritten: an interventional study

**DOI:** 10.1186/s12911-022-01835-4

**Published:** 2022-04-08

**Authors:** Sahar Peivandi, Leila Ahmadian, Jamileh Farokhzadian, Yunes Jahani

**Affiliations:** 1 Department of Health Information Sciences, Faculty of Management and Medical Information Sciences, Kerman University of Medical Sciences, Kerman, Iran; 2 Nursing Research Center, Kerman University of Medical Sciences, Kerman, Iran; 3 Modeling in Health Research Center, Institute for Futures Studies in Health, Kerman University of Medical Sciences, Kerman, Iran

**Keywords:** Documentation, Electronic medical record, Errors, Speech recognition software, Voice recognition, Nurses, Nursing note, Paper note

## Abstract

**Background:**

Despite the rapid expansion of electronic health records, the use of computer mouse and keyboard, challenges the data entry into these systems. Speech recognition software is one of the substitutes for the mouse and keyboard. The objective of this study was to evaluate the use of online and offline speech recognition software on spelling errors in nursing reports and to compare them with errors in handwritten reports.

**Methods:**

For this study, online and offline speech recognition software were selected and customized based on unrecognized terms by these softwares. Two groups of 35 nurses provided the admission notes of hospitalized patients upon their arrival using three data entry methods (using the handwritten method or two types of speech recognition software). After at least a month, they created the same reports using the other methods. The number of spelling errors in each method was determined. These errors were compared between the paper method and the two electronic methods before and after the correction of errors.

**Results:**

The lowest accuracy was related to online software with 96.4% and accuracy. On the average per report, the online method 6.76, and the offline method 4.56 generated more errors than the paper method. After correcting the errors by the participants, the number of errors in the online reports decreased by 94.75% and the number of errors in the offline reports decreased by 97.20%. The highest number of reports with errors was related to reports created by online software.

**Conclusion:**

Although two software had relatively high accuracy, they created more errors than the paper method that can be lowered by optimizing and upgrading these softwares. The results showed that error correction by users significantly reduced the documentation errors caused by the software.

## Background

Clinical documentation is the most important source of information about the patient and is used to assess the patient's problems and to manage his clinical conditions [1, 2]. These documents are also important in matters of patient safety [3–7], quality assurance [7–9], legal processes [7, 10, 11], financial issues [5, 10–12], and medical education [13–15]. Paper documents have several problems, including illegibility and incompleteness [16]. Studies have shown that illegible handwriting can increase medication errors by 75% [17, 18]. To overcome these problems, electronic medical records are being developed and replace paper documents worldwide. Because of the advantages of the electronic health record including better readability and accessibility of data [19], health organizations have become more interested in using computers to create records than making paper records. Despite the advantages of electronic records, they require more eye-hand coordination due to the simultaneous use of mouse and keyboard while looking at the computer screen [20]. The use of a mouse and keyboard is also difficult for users with low typing skills or speed [21].

One of the data entry methods is the speech recognition method. This method, which converts audio to text can be an alternative to typing by mouse and keyboard and reduce user fatigue. In recent years, the use of this software has increased due to its ease of use among health care providers [22, 23]. Previous studies have shown that this software has been used successfully for recording radiology reports [24]. Today, physicians and nurses are commonly using speech recognition software for documentation [25]. When using this software to create nursing reports, due to access to oral data, less information is lost and misinterpretations are reduced [26]. Also in a study, this software increased the quality of nursing reports because of direct and on-time recording of data as opposed to handwritten reports which may be recorded hours later [27]. However, few studies have examined the effect of using speech recognition software on nursing reports [22, 23, 25, 26, 28]. In addition, to our knowledge, none of the studies has compared the number of errors between paper nursing reports and electronic reports created by different types of speech recognition software. The objective of this study was evaluation and comparison of errors including spelling errors, missing words, and added words (words typed without being uttered by the user) on nursing notes created by online and offline speech recognition technology and handwritten.

## Methods

This interventional study was carried out on nurses working in inpatient wards of three educational hospitals affiliated to Kerman University of Medical Sciences. Nurses were randomly selected from 10 clinical wards of three hospitals including gynecology, gastroenterology, midwifery, general surgery, orthopedics, ENT surgery, reconstructive surgery, cardiac surgery, eye surgery, and cardiology. Intensive care units and operating room wards were not included in the study due to the difference in nursing reports between these wards and other wards, as well as their high workload compared to other wards resulting in lower cooperation of their nurses. Criteria for the inclusion of nurses in the study were having at least 6 months of work experience in the studied wards and participation in generating daily nursing reports.

A sample of 70 nurses was recruited to participate and evaluate the errors generated by speech recognition software. This sample size was calculated based on the data of a previous study that was conducted to examine the accuracy of the medical report generated by the speech recognition system and the experience of physicians in hospitals [29], using the following formula and considering (µ1 − µ2 = 10) d = 10 and (1 − β = 0.80).

$$n = \frac{{2\left( {z_{{1 - \frac{\alpha }{2}}} + z_{1 - \beta } } \right)^{2} \sigma^{2} }}{{d^{2} }}$$

The significant level was 0.05.

We used a crossover study design to discard the learning effect, and divided participants into two groups of 35 nurses randomly. The participants in each group were asked to provide the admission nursing note upon arrival of one of the recently hospitalized patients using the paper or two online and offline speech recognition methods. The reason for selecting admission notes was that these notes have identical structures in all wards, and are the most comprehensive nursing notes for each patient. After at least 1 month, nurses were asked to provide the same reports using the opposite method. All reports were created in the real workplace of nurses in the inpatient wards. To somewhat manage working place noises, reports were not created at patient’s bedside and all reports were created in nursing stations.

### Selection and customization of speech recognition software

In this study, the professional version (3.2) of Nevisa, an offline speech recognition software, and Speechtexter, an online speech recognition software were used. Both of these software programs are compatible with Windows operating system and support the Persian language. It is also possible to add an unlimited number of words to them. To use the offline software, a hardware lock was required to connect to the system. An external sound card (to transmit sound transparently to software) and Andrea (NC-8) Head Mounted microphone was also used.

Since these two software only use general non-specialized terms and do not support the recognition of medical terms, terms that could not be recognized by them were added to the software. For this purpose, on average 10 patient records from each of the studied wards were randomly retrieved. From which 80 records were selected and their admission nursing notes were reviewed. Then, one of the researchers read each of these reports verbally once to the online speech recognition software and once to the offline software. The terms that could not be recognized by the software were added to their dictionary.

Nevisa was offline and required training of the user's voice to the software. To do this, each user was asked to read 120 predefined sentences determined by the system developers. To train to nurses’ voices for the software reading at least 40 of the 120 sentences is required, as defined by the developer. This took about 20–30 min per user.

Speechtexter was online and did not require user voice training. In order to create nursing reports by speech recognition software, an electronic form of the nursing report was created using Access software.

When the reports were created by the participants, they were asked to review the reports and correct any potential errors. Then one of the researchers reviewed the initial reports (pre-correction reports) and identified their errors. The reports and the errors were checked and confirmed by a nurse who was not among the participant and gained knowledge about how to evaluate the reports and what to considered as error. After it, the same researcher reviewed the corrected reports again and identified their errors.

### Data analysis

The collected data were analyzed using Minitab 18. Since we used a crossover design, a nested ANOVA was used to compare the number of errors among three methods of documenting nursing reports. Moreover, in order to find the difference between the number of errors among the three methods, the Tukey statistical test was used. Moreover, the analysis of the values related to the accuracy of each method was expressed descriptively and as a percentage.

## Results

A total of 70 nurses participated in the study. Most of the participants were female (n = 60). The age range of the participants was from 22 to 45 years. Based on the review of the nursing reports in inpatient records, a total of 521 words were added to the offline and 695 words to the online software. On average, 6 words were added to the offline and 8 words to the online software per report. The accuracy of nursing reports generated by the online and offline software was 96.4% and 97.52% respectively. After correcting the errors by nurses, the accuracy of online software reached 99.81% and the accuracy of offline software reached 99.93%. Table 1 shows the descriptive information obtained from this step:

**Table 1:** Number of reports with errors in three methods of documenting nursing reports

Methods of documentation	Number of errors (%)			
	Reports without error	Reports with an error	Reports with more than one error	Total
Paper	67 (95.71)	3 (4.28)	0 (0)	70
Online	1 (1.43)	3 (4.28)	66 (94.28)	70
Offline	2 (2.86)	4 (5.71)	64 (91.43)	70

Errors were found in three out of the 70 reports generated by the paper method. From the reports generated by online and offline software, 69 and 68 reports had errors, respectively. There was more than one error in 94% of reports created with online and in 91% of reports created with offline software. On average, the rate of errors per report was 0.04 errors in the handwritten reports, 6.80 errors in online reports, and 4.60 errors in offline reports.

The results of nested ANOVA to compare the three methods of documenting nursing reports in terms of the number of errors are summarized in Table 2:

**Table 2:** Comparison of errors made in the three methods of documenting nursing reports

Source	DF^a^	Adj SS^b^	Adj MS^c^	*F*-Value	*P* Value
Period	1	8.57	8.571	1.27	0.26
Sequence^d^	1	12.34	12.343	1.24	0.26
Interven^e^	2	1662.89	831.443	123.20	0.00^*^
Nurse(Sequence)	68	701.09	10.310	1.53	0.01
Error	137	924.54	6.748		
Total	209	3319.76			

^*^P < .05

^a^Number of degrees of freedom

^b^Adjusted sum of squares

^c^Adjusted mean squares

^d^Consisting of two groups, each group of 35 participants in one course has created its reports by paper method and in another course by electronic method

^e^Three intervention methods include paper method, online software method, offline software method

The results of this test showed statistically significant differences between these three methods (*P* = 0.00). To examine the difference between the numbers of errors made by the three methods, the Tukey test was used and all three methods were compared with each other in pairs. The results of this test are shown in Table 3:

**Table 3:** The differences of the average number of errors per report among the three methods of documenting nursing reports

Compared methods	Mean ± SD	CI 95%	*T*-Value	*P* Value
Online and paper	6.76 ± 0.44	(5.72, 7.80)	15.39	0.00*
Offline and paper	4.56 ± 0.44	(3.52, 5.60)	10.38	0.00*
Offline and online	− 2.20 ± 0.44	(− 3.24, − 1.16)	− 5.01	0.00*

^*^P < 0.05

As shown in Table 3, there was a statistically significant difference between all three methods of documenting nursing reports in terms of the number of errors (*P* = 0.00). On average, the number of errors per report in the online method was 6.76 errors, and in the offline method 4.56 errors higher than in the paper method. The number of errors in online reports was on average 2.2 higher than in the offline reports. The highest error rate was generated by the online speech recognition method with an average of 6.8 errors per report and the lowest rate was generated by the paper method with an average of 0.04 errors per report.

The results showed that only 4% of handwritten reports had errors, while 98% of online reports and 97% of offline reports had at least one error.

Among the online reports, only 1 report was error-free, and the maximum number of errors was 9 in one report. Also, the lowest number of errors in the offline reports was 0 and the highest number was 16 in one report. In general, the paper method had fewer errors than the electronic methods.

After correcting the errors by the participants, the number of errors in the online reports decreased by 94.75% to 25 errors in the total reports (on average 0.35 errors per report) and the number of errors in the offline reports decreased by 97.20% to 9 errors (on average 0.12 errors per report). Also, the total number of erroneous reports after correction were reduced to 20 reports in the online method (28.57% all online reports) and to 7 reports in the offline method (10% of all offline reports).

## Discussion

The present study was conducted to evaluate speech recognition software on the number of spelling errors in admission nursing notes. The results showed a significant difference among the three methods of documenting nursing reports in terms of the number of errors. Reports created with online speech recognition software had the highest and handwritten reports had the lowest number of errors. The results of this study showed that the error rate was significantly reduced after editing the reports created by the two speech recognition software. Also, the number of errors in online reports was significantly higher than in the offline reports. One of the reasons for the higher number of errors in online reports compared to offline reports is the dependence of online software on the speed of the Internet and consequently failure to type some words when slowing down the Internet. This was the cause of many errors related to this software.

Consistent with our results, other studies [24, 30, 31] have shown that the number of errors in radiology reports created by speech recognition software was higher than in paper-based radiology reports. Zhou et al. [32] also examined the effect of this software on medical reports and, similar to our results, reported that the error rate after correction of reports is greatly reduced. However, in their study, after corrections 40% of reports still had errors while in our study this number decrease to 28% for online reports and to 10% for offline reports. Errors in medical records can affect the quality of patient care [33–36] and reduce productivity [24]. According to the literature, among the studies conducted on speech recognition software, only one study on pathology reports indicated a reduction in the errors of software reports compared to handwritten reports [37]. The error may occur due to several factors such as the type of software, user accent, and experience, speed of speaking, and environmental factors such as crowded conditions [29]. The amount of error can also be related to the complexity of the report [30]. The use of common terms in recording reports can reduce documentation errors. In addition, using macros and new versions of speech recognition software can reduce these errors [38]. Use of artificial intelligence in this software reduces the number of errors over time due to software learning [37]. One of the most important reasons for higher errors in the speech recognition method compared to the handwritten method in this study was the use of general and non-specialized software. At the beginning and before adding medical words, these software programs were unable to recognize medical terms. Despite adding a large number of medical terms for customizations, these software programs could not recognize some of these terms. Another reason was the use of Persian speech recognition software, which has been developed recently and still need time to improve their diagnostic engines. In addition, Persian language users do not have much experience working with speech recognition software.

Our results showed that the accuracies of online (96%) and offline methods (97%), are lower than the accuracy of the handwritten method (99.99%). Congruent to our results Hammana et al.[24] found that the accuracy of speech recognition software for generating radiology reports is lower than the handwritten method. The accuracy of speech recognition software has been reported between 80 and 98% by other studies [39–45]. Speech disorders such as stuttering, pauses, and interruptions, which are part of human nature, are among the reasons for the lower accuracy of speech recognition software compared to traditional and handwritten methods. The sounds emerge due to these disorders are interpreted by the software as words [24]. Speech recognition software has the potential to be improved in the future and consequently reduce errors, just as the development of this software over the past 20 years has significantly reduced errors compared to the original speech recognition software [46].

Despite studies investigating the impact of speech recognition technology on nursing reports, so far no research has used this software in the real world and at the point of care. This research is the first study for evaluation of this software on nursing reports by creating real reports related to patients. The results of this study can help health policymakers to make proper decisions for replacing the handwritten method with the speech recognition method and to select the appropriate software. These results also provide a broader perspective to the developers of these systems to carefully address their shortcomings and design accurate software. The results of this study can be used to develop medical speech recognition software. This study was conducted to evaluate speech recognition software on admission nursing notes due to the uniform structure of these reports in all wards. It is suggested that in future studies, the impact of this software on other reports be examined.

### Limitations

This study had four limitations. First, due to the lack of medical speech recognition software with Persian language support, software with general and non-specialized terms recognition was used. To eliminate this limitation, a large number of medical records were reviewed to extract specialized terms and add them to the software. Moreover, some nurses used to apply abbreviations and shorthand notations to record their notes, that need to be added to the software. Second, to work with offline software, it was necessary to train the user's voice to the software, while some nurses were reluctant to participate in the research due to a shortage of time. Therefore, this was done during the hours of the day when there was less workload in the study area. Also, nurses who did not want to spend their time doing this were not included in the study. Third, since the use of online software requires the use of the Internet, due to the low-speed Internet in some wards, the software had problems in recognizing some terms leading to errors. Fourth, although it was better to compare speech recognition technology with mouse and keyboard, but in Iran, medical records are still recorded manually and on paper. Forth, one of the limitations of this study was that users have different technological literacy and each of them needs different time to reach an acceptable level. This can affect the results. Moreover, our results may be affected by proficiency of the users as users have never worked with this software before.

## Conclusion

The present study showed that although the use of speech recognition software for recording nursing reports may lead to errors, the review of reports by users could greatly result in the correction of errors. In addition, the results showed that offline software has a lower rate of errors in recording reports than online software. This study provided a good insight for health policymakers, designers, and developers of these systems to select and develop appropriate and efficient software.

## Data Availability

Since, the research data set include more data beyond the data analyzed and published in this study, and the authors still working on other manuscripts based on this data set, the data generated and analyzed during this study are available from the corresponding author on reasonable request.
